# Association between Cutaneous Nevi and Breast Cancer in the Nurses' Health Study: A Prospective Cohort Study

**DOI:** 10.1371/journal.pmed.1001659

**Published:** 2014-06-10

**Authors:** Mingfeng Zhang, Xuehong Zhang, Abrar A. Qureshi, A. Heather Eliassen, Susan E. Hankinson, Jiali Han

**Affiliations:** 1 Department of Dermatology, Brigham and Women's Hospital and Harvard Medical School, Boston, Massachusetts, United States of America; 2 Channing Division of Network Medicine, Department of Medicine, Brigham and Women's Hospital and Harvard Medical School, Boston, Massachusetts, United States of America; 3 Department of Dermatology, Rhode Island Hospital, Warren Alpert Medical School, Brown University, Providence, Rhode Island, United States of America; 4 Department of Epidemiology, Harvard School of Public Health, Boston, Massachusetts, United States of America; 5 Division of Biostatistics and Epidemiology, School of Public Health and Health Sciences, University of Massachusetts, Amherst, Massachusetts, United States of America; 6 Department of Epidemiology, Richard M. Fairbanks School of Public Health, Indiana University, Indianapolis, Indiana, United States of America; 7 Melvin and Bren Simon Cancer Center, Indiana University, Indianapolis, Indiana, United States of America; 8 Department of Dermatology, School of Medicine, Indiana University, Indianapolis, Indiana, United States of America; 9 Department of Epidemiology, Tianjin Medical University Cancer Institute and Hospital, Tianjin, China; Medical School, King's College London, United Kingdom

## Abstract

**Background:**

Cutaneous nevi are suggested to be hormone-related. We hypothesized that the number of cutaneous nevi might be a phenotypic marker of plasma hormone levels and predict subsequent breast cancer risk.

**Methods and Findings:**

We followed 74,523 female nurses for 24 y (1986–2010) in the Nurses' Health Study and estimate the relative risk of breast cancer according to the number of cutaneous nevi. We adjusted for the known breast cancer risk factors in the models. During follow-up, a total of 5,483 invasive breast cancer cases were diagnosed. Compared to women with no nevi, women with more cutaneous nevi had higher risks of breast cancer (multivariable-adjusted hazard ratio, 1.04, 95% confidence interval [CI], 0.98–1.10 for 1–5 nevi; 1.15, 95% CI, 1.00–1.31 for 6–14 nevi, and 1.35, 95% CI, 1.04–1.74 for 15 or more nevi; *p* for continuous trend = 0.003). Over 24 y of follow-up, the absolute risk of developing breast cancer increased from 8.48% for women without cutaneous nevi to 8.82% (95% CI, 8.31%–9.33%) for women with 1–5 nevi, 9.75% (95% CI, 8.48%–11.11%) for women with 6–14 nevi, and 11.4% (95% CI, 8.82%–14.76%) for women with 15 or more nevi. The number of cutaneous nevi was associated with increased risk of breast cancer only among estrogen receptor (ER)–positive tumors (multivariable-adjusted hazard ratio per five nevi, 1.09, 95% CI, 1.02–1.16 for ER+/progesterone receptor [PR]–positive tumors; 1.08, 95% CI, 0.94–1.24 for ER+/PR− tumors; and 0.99, 95% CI, 0.86–1.15 for ER−/PR− tumors). Additionally, we tested plasma hormone levels according to the number of cutaneous nevi among a subgroup of postmenopausal women without postmenopausal hormone use (*n* = 611). Postmenopausal women with six or more nevi had a 45.5% higher level of free estradiol and a 47.4% higher level of free testosterone compared to those with no nevi (*p* for trend = 0.001 for both). Among a subgroup of 362 breast cancer cases and 611 matched controls with plasma hormone measurements, the multivariable-adjusted odds ratio for every five nevi attenuated from 1.25 (95% CI, 0.89–1.74) to 1.16 (95% CI, 0.83–1.64) after adjusting for plasma hormone levels. Key limitations in this study are that cutaneous nevi were self-counted in our cohort and that the study was conducted in white individuals, and thus the findings do not necessarily apply to other populations.

**Conclusions:**

Our results suggest that the number of cutaneous nevi may reflect plasma hormone levels and predict breast cancer risk independently of previously known factors.

*Please see later in the article for the Editors' Summary*

## Abstract 2

Using data from the Nurses' Health Study, Jiali Han and colleagues examine the association between number of cutaneous nevi and the risk for breast cancer.

*Please see later in the article for the Editors' Summary*

## Editors' Summary

**Background:**

One woman in eight will develop breast cancer during her lifetime. Breast cancer begins when cells in the breast acquire genetic changes that allow them to divide uncontrollably (which leads to the formation of a lump in the breast) and to move around the body (metastasize). The treatment of breast cancer, which is diagnosed using mammography (a breast X-ray) or manual breast examination and biopsy, usually involves surgery to remove the lump, or the whole breast (mastectomy) if the cancer has started to metastasize. After surgery, women often receive chemotherapy or radiotherapy to kill any remaining cancer cells and may also be given drugs that block the action of estrogen and progesterone, female sex hormones that stimulate the growth of some breast cancer cells. Globally, half a million women die from breast cancer each year. However, in developed countries, nearly 90% of women affected by breast cancer are still alive five years after diagnosis.

**Why Was This Study Done?:**

Several sex hormone–related factors affect breast cancer risk, including at what age a woman has her first child (pregnancy alters sex hormone levels) and her age at menopause, when estrogen levels normally drop. Moreover, postmenopausal women with high circulating levels of estrogen and testosterone (a male sex hormone) have an increased breast cancer risk. Interestingly, moles (nevi)—dark skin blemishes that are a risk factor for the development of melanoma, a type of skin cancer—often darken or enlarge during pregnancy. Might the number of nevi be a marker of hormone levels, and could nevi counts therefore be used to predict an individual's risk of breast cancer? In this prospective cohort study, the researchers look for an association between number of nevi and breast cancer risk among participants in the US Nurses' Health Study (NHS). A prospective cohort study enrolls a group of people, determines their baseline characteristics, and follows them over time to see which characteristics are associated with the development of certain diseases. The NHS, which enrolled 121,700 female nurses aged 30–55 years in 1976, is studying risk factors for cancer and other chronic diseases in women.

**What Did the Researchers Do and Find?:**

In 1986, nearly 75,000 NHS participants (all of whom were white) reported how many nevi they had on their left arm. Over the next 24 years, 5,483 invasive breast cancers were diagnosed in these women. Compared to women with no nevi, women with increasing numbers of nevi had a higher risk of breast cancer after adjustment for known breast cancer risk factors. Specifically, among women with 1–5 nevi, the hazard ratio (HR) for breast cancer was 1.04, whereas among women with 15 or more nevi the HR was 1.35. An HR compares how often a particular event occurs in two groups with different characteristics; an HR greater than one indicates that a specific characteristic is associated with an increased risk of the event. Over 24 years of follow-up, the absolute risk of developing breast cancer was 8.48% in women with no nevi but 11.4% for women with 15 or more nevi. Notably, postmenopausal women with six or more nevi had higher blood levels of estrogen and testosterone than women with no nevi. Finally, in a subgroup analysis, the association between number of nevi and breast cancer risk disappeared after adjustment for hormone levels.

**What Do These Findings Mean?:**

These findings support the hypothesis that the number of nevi reflects sex hormone levels in women and may predict breast cancer risk. Notably, they show that the association between breast cancer risk and nevus number was independent of known risk factors for breast cancer, and that the risk of breast cancer increased with the number of nevi in a dose-dependent manner. These findings also suggest that a hormonal mechanism underlies the association between nevus number and breast cancer risk. Because this study involved only white participants, these findings may not apply to non-white women. Moreover, the use of self-reported data on nevus numbers may affect the accuracy of these findings. Finally, because this study is observational, these findings are insufficient to support any changes in clinical recommendations for breast cancer screening or diagnosis. Nevertheless, these data and those in an independent *PLOS Medicine* Research Article by Kvaskoff et al. support the need for further investigation of the association between nevi and breast cancer risk and of the mechanisms underlying this relationship.

**Additional Information:**

Please access these websites via the online version of this summary at http://dx.doi.org/10.1371/journal.pmed.1001659.

- An independent *PLOS Medicine* Research Article by Kvaskoff et al. also investigates the relationship between nevi and breast cancer risk
- The US National Cancer Institute provides comprehensive information about cancer (in English and Spanish), including detailed information for patients and professionals about breast cancer; it also has a fact sheet on moles
- Cancer Research UK, a not-for profit organization, provides information about cancer, including detailed information on breast cancer
- The UK National Health Service Choices website has information and personal stories about breast cancer; the not-for profit organization Healthtalkonline also provides personal stories about dealing with breast cancer
- More information about the Nurses' Health Study is available

## Introduction

Epidemiologic studies have identified a number of hormone-related risk factors for breast cancer, such as age at menarche, age at first birth, and age at menopause [1],[2]. Further, increased circulating levels of estradiol and testosterone in postmenopausal women are consistently associated with increased breast cancer risk [3]–[5]. It has been noticed that melanocytic nevi commonly darken and/or enlarge during pregnancy, suggesting a possible linkage between nevi and hormones [6],[7]. Therefore, we hypothesized that the number of cutaneous nevi might be a phenotypic marker of plasma hormone levels, and thus may also predict the risk of subsequent breast cancer. We conducted a prospective analysis in the Nurses' Health Study (NHS), a large cohort study (121,700 female nurses) with information on self-counted number of cutaneous nevi. We followed the participants from 1986, when information on the number of cutaneous nevi was collected, up to 2010.

## Methods

### Ethics Statement

The protocol for this study was approved by the Institutional Review Board at Brigham and Women's Hospital and the Harvard School of Public Health. All of the participants provided informed consent.

### Study Population

The NHS cohort was established in 1976, when 121,700 female registered nurses aged 30 to 55 y completed a baseline questionnaire including items on risk factors for cancer and cardiovascular disease. Updated risk factor information and disease development were obtained by follow-up questionnaires every 2 y. Follow-up has been extremely high, with only 4.4% of person-time lost to follow-up. Details of this cohort have been described previously [8].

The baseline was 1986 for this study, when participants reported self-counted number of cutaneous nevi. From the baseline cohort of 101,516 women in the 1986 follow-up cycle, after excluding those who developed cancer before 1986 (*n* = 7,157), those who did not report the number of cutaneous nevi (*n* = 18,020), and those who were non-white (*n* = 1,816), 74,523 women were included in the analysis.

### Exposure Data

In the baseline questionnaire in 1986, the NHS cohort participants reported numbers of cutaneous nevi on their left arms from shoulder to wrist of ≥3 mm diameter size using the categories of none, 1–2, 3–5, 6–9, 10–14, 15–20, and more than 20. Information on body weight, physical activity, multivitamin use, smoking status, menopausal status, age at menopause, postmenopausal hormone (PMH) use, personal history of benign breast disease (confirmed by breast biopsy), and age at first birth and parity was collected in the baseline questionnaire and was updated in the follow-up biennial questionnaires. Information on alcohol consumption was collected in the follow-up questionnaires every 4 y beginning in 1986. Information on family history of breast cancer was collected in the follow-up questionnaires every 4 y beginning in 1988. Information on age at menarche, height, and body weight at age 18 was collected in 1976. Body mass index was calculated from body weight and height. Information on hair color (categorized into red, blond, light brown, dark brown, and black) and tanning ability (categorized into particularly none, light tan, average tan, and deep tan) was collected in 1982. Information on outdoor sun exposure was collected in the 2006 questionnaire. We asked how many hours per week each participant spent outdoor in direct sunlight in the middle of the day during summer during high school and at ages 25–35, 36–59, and 60–65 y. UV flux for each study participant was estimated based on residential history according to detailed methods documented previously [9]. Briefly, the potential cumulative UV flux that a participant could have received over a period of time was estimated by summing the annual UV flux data over the follow-up period.

### Identification of Breast Cancer Cases

Participants reported new breast cancer diagnoses biennially. For women who did not respond to the questionnaires, we search the National Death Index routinely for deaths, and the last search was conducted in December 2010. We asked all women who reported breast cancer (or next of kin for those who died) for permission to review the pertinent medical records for confirmation. Information on date of diagnosis was collected from the medical records as well. Pathology reports, obtained in 96% of the cases, showed a 99.4% confirmation rate [8]. Eligible cases consisted of women with incident breast cancer diagnosed any time between baseline and June 2010, with no previously diagnosed cancer. Only pathologically confirmed invasive cases were included in the primary analysis. We included carcinomas in situ in a secondary analysis. The estrogen receptor (ER) and progesterone receptor (PR) status of tumors was abstracted from pathology reports.

### Plasma Hormone Study

A subgroup of NHS participants provided plasma samples between 1989 and 1990. The plasma hormone analysis used 611 controls from a nested case-control study of breast cancer in the NHS [10]. The controls in this study were women who, at blood collection, were postmenopausal and had not used PMHs for at least 3 mo. Hormone assay methods have been described previously [11],[12]. We measured the levels of estradiol, testosterone, sex hormone–binding globulin, and dehydroepiandrosterone sulfate. Free estradiol and free testosterone were calculated by the law of mass action [13]. Within-batch laboratory coefficients of variation ranged from 8% to 12%. Details of sample collection and measurement have been described previously [10]. In addition, we conducted a case-control study among the 362 breast cancer cases and 611 matched controls in this subgroup to adjust for plasma hormone levels in the association between nevus count and breast cancer.

### Statistical Analysis

Participants contributed person-time from the baseline in June 1986 to the end of follow-up. Accumulation of follow-up time ceased at the diagnosis of any type of cancer, death, or the end of follow-up, whichever came earliest. We used age- and multivariable-adjusted Cox proportional hazards models to calculate the hazard ratios (HRs) and 95% confidence intervals (CIs) of breast cancer according to the number of cutaneous nevi. Based on the categorical information from the questionnaire, we grouped women into four categories by number of cutaneous nevi: none, 1–5, 6–14, and 15 or more nevi, and compared each of the last three groups with the group reporting no nevi. To test for a linear trend, we modeled the number of cutaneous nevi as a continuous variable by using the median value of each category and the lower bound of the top category. In multivariable regression models, we adjusted for age and additional covariates representing possible confounders and known breast cancer risk factors, including menopausal status, age at menarche, parity and age at first birth, body mass index, body mass index at age 18 y, height, physical activity, multivitamin use, family history of breast cancer in a first-degree relative, cigarette smoking, alcohol consumption, and self-report of benign breast disease. All variables except age at menarche and body mass index at age 18 y were updated throughout follow-up. For postmenopausal women, terms were also included for duration of menopause and PMH use. To assess the proportionality assumption, we created interactive terms for the main predictors and time to event (nevi×time) in the larger model, and compared the smaller model without any time-dependent main predictors to the larger model that included all of the time-dependent main predictors. The test was carried out by a Wald statistic with one degree of freedom and chi-square distribution under the null hypothesis. The population attributable risk (PAR) was calculated based on the formula PAR = *P*_e_ (HR_e_−1)/(1+*P*_e_ [HR_e_−1]), where *P*_e_ is the prevalence of the exposure, and HR_e_ is the HR of disease due to the exposure. Tests for interaction were performed using the Wald test for the cross-product interaction term. In analysis of plasma hormones, because of the smaller sample size, we collapsed the top nevus count categories to one: those with six or more nevi. We used generalized linear models to compare mean hormone levels across subgroups with different numbers of cutaneous nevi (none, 1–2, 3–5, 6+ nevi on arm). We controlled for fasting status (<8 h, ≥8 h), time of day (24-h clock: <8, 8–12, 13–24), age at blood draw (continuous), season of blood draw (May to October, other months), alcohol intake (continuous), smoking status (never, ever), and body mass index at blood draw (continuous). For the trend test, we coded the number of cutaneous nevi as a continuous variable as described above. All of the statistical analyses were carried out using Statistical Analysis System software (version 9.1.3; SAS Institute). All *p*-values were two-sided.

## Results

Among 74,523 women followed from 1986 until 2010, with 1.5 million person-years, 5,483 cases of invasive breast cancer were documented. In Table 1, we present the basic characteristics of the study population according to the number of cutaneous nevi. Women with more cutaneous nevi were more likely to have hypertension and history of benign breast disease. Among postmenopausal women, those with more cutaneous nevi were more likely to be current PMH users. Current smokers, heavier alcohol consumers, and nulliparous women tended to have fewer cutaneous nevi. Other breast cancer risk factors were distributed fairly evenly across the groups of different numbers of cutaneous nevi. The baseline characteristics were similar between those with and without information on cutaneous nevi (Table S1).

**Table 1:** Baseline characteristics of women according to the self-reported number of cutaneous nevi.

Characteristic	Number of Cutaneous Nevi				*p*-Value
	None (*n* = 47,052)	1–5 (*n* = 24,017)	6–14 (*n* = 2,789)	15+ (*n* = 665)	
Mean age, y (SD)	52.7 (7.2)	52.0 (7.1)	51.9 (7.1)	52.0 (7.2)	<0.01
Body mass index, kg/m^2^ (SD)	25.1 (4.7)	25.6 (4.9)	26.5 (5.5)	26.8 (5.7)	<0.01
Body mass index at age 18 y, kg/m^2^ (SD)	21.3 (3.0)	21.4 (3.0)	21.7 (3.2)	22.0 (3.6)	<0.01
Height in 1976, m (SD)	64.4 (3.1)	64.5 (3.2)	64.7 (3.0)	64.8 (2.5)	<0.01
Physical activity, MET h/wk (SD)	14.1 (20.8)	14.0 (20.7)	13.7 (19.5)	14.8 (24.5)	0.56
Multivitamin use, percent	43.0	43.3	42.6	44.3	0.79
Current smoker, percent	22.2	20.4	20.0	19.2	<0.01
Alcohol consumption, g/d (SD)	6.3 (11.0)	6.1 (10.4)	5.8 (9.8)	5.0 (8.5)	<0.01
Age at menarche ≤12 y, percent	49.3	48.8	49.6	49.1	0.95
Nulliparous, percent	6.6	6.7	7.5	7.7	0.20
Age at first birth, y (SD)	29.3 (17.2)	29.3 (17.2)	30.2 (18.7)	30.1 (18.3)	0.08
History of benign breast disease, percent	14.9	16.5	16.9	19.8	<0.01
Family history of breast cancer, percent	10.4	10.8	11.2	9.2	0.52
Postmenopausal, percent	61.0	61.0	60.3	61.2	<0.01
Duration of menopause among postmenopausal women, y (SD)	10.2 (6.4)	10.2 (6.7)	10.5 (6.8)	10.7 (6.9)	0.24
Current hormone use among postmenopausal women, percent	26.7	27.6	28.4	30.8	<0.01

Based on information collected in the 1986 questionnaire unless specified otherwise.

MET, metabolic equivalent of task; SD, standard deviation.

Women with more cutaneous nevi had a significantly increased risk of developing subsequent breast cancer (Table 2). After adjusting for previously known breast cancer risk factors, the HR was 1.04 (95% CI, 0.98–1.10) for women with 1–5 nevi, 1.15 (95% CI, 1.00–1.31) for women with 6–14 nevi, and 1.35 (95% CI, 1.04–1.74) for women with 15 or more nevi, compared to those without cutaneous nevi. Over 24 y of follow-up, the absolute risk of developing breast cancer increased from 8.48% for women without cutaneous nevi to 8.82% (95% CI, 8.31%–9.33%) for women with 1–5 nevi, 9.75% (95% CI, 8.48%–11.11%) for women with 6–14 nevi, and 11.4% (95% CI, 8.82%–14.76%) for women with 15 or more nevi. The trend of this increased risk was significant (*p* = 0.003), and every five additional nevi were associated with an 8% increase in breast cancer risk (multivariable-adjusted HR, 1.08, 95% CI, 1.03–1.13). These results were not substantially changed by the additional inclusion of 1,084 in situ breast cancer cases. The distribution of nevus count in our study population was positively skewed (*n* = 47,052 for none, 18,032 for 1–2 nevi, 5,985 for 3–5 nevi, 1,969 for 6–9 nevi, 820 for 10–14 nevi, 330 for 15–20 nevi, and 325 for more than 20 nevi). Thus, we considered that the risk estimates for the categorical groups were more appropriate than those for the continuous count of nevi. We examined the proportional hazard assumption of nevus count, and it was valid by statistical tests (*p* = 0.55).

**Table 2:** The number of cutaneous nevi and breast cancer risk by estrogen and progesterone receptor status.

Number of Cutaneous Nevi	All Breast Cancer			ER+/PR+ Cancer		ER+/PR− Cancer		ER−/PR− Cancer	
	Cases	Age-Adjusted HR (95% CI)	Multivariable-Adjusted HR (95% CI)^a^	Cases	Multivariable-Adjusted HR (95% CI)^a^	Cases	Multivariable-Adjusted HR (95% CI)^a^	Cases	Multivariable-Adjusted HR (95% CI)^a^
None	3,382	Ref	Ref	1,972	Ref	414	Ref	442	Ref
1–5	1,809	1.07 (1.01, 1.13)	1.04 (0.98, 1.10)	1,092	1.06 (0.99, 1.15)	201	0.96 (0.81, 1.14)	208	0.90 (0.77, 1.07)
6–14	231	1.20 (1.05, 1.37)	1.15 (1.00, 1.31)	138	1.17 (0.98, 1.39)	28	1.19 (0.81, 1.76)	24	0.89 (0.59, 1.35)
15+	61	1.36 (1.06, 1.76)	1.35 (1.04, 1.74)	36	1.33 (0.95, 1.85)	8	1.57 (0.78, 3.18)	9	1.59 (0.82, 3.08)
HR per five nevi	5,483	1.09 (1.04, 1.15)	1.08 (1.03, 1.13)	3,238	1.09 (1.02, 1.16)	651	1.08 (0.94, 1.24)	683	0.99 (0.86, 1.15)
*p* for trend		<0.001	0.003		0.01		0.28		0.94

a Adjusted for age, menopausal status, age at menarche, parity and age at first birth, body mass index, body mass index at age 18 y, height, physical activities, multivitamin use, family history of breast cancer in a first-degree relative, cigarette smoking, alcohol consumption, self-report of benign breast disease, as well as duration of menopause and hormone use among postmenopausal women.

We hypothesized that the number of cutaneous nevi could be a biomarker of plasma hormone levels, so we further evaluated this association by ER/PR status of the tumors. Consistent with this hypothesis, we observed that the number of nevi seemed to be associated only with the risk of ER-positive cancers (multivariable-adjusted HR, 1.09, 95% CI, 1.02–1.16 for ER+/PR+ tumors; 1.08, 95% CI, 0.94–1.24 for ER+/PR− tumors; and 0.99, 95% CI, 0.86–1.15 for ER−/PR− tumors, for every five additional nevi; Table 2). The heterogeneity *p*-value for this difference was 0.24.

Furthermore, we tested the association between the number of cutaneous nevi and plasma hormone levels among a subgroup of 611 postmenopausal participants with plasma hormone measurements and without PMH use. We found that women with more nevi had higher levels of estradiol and testosterone (*p* for trend = 0.02 for estradiol and 0.06 for testosterone; Table 3). The associations with free estradiol and free testosterone were highly significant (45.5% higher level of free estradiol and 47.4% higher level of free testosterone among women with six or more nevi compared to those with no nevi, both *p* for trend = 0.001; Table 3). Both of these associations remained significant after adjusting for multiple comparisons (0.05/6 = 0.008 for each of the six hormonal markers). The correlations between nevus count and hormonal levels were moderate (*r* ^2^ = 0.30 for free estradiol and 0.09 for free testosterone).

**Table 3:** The number of cutaneous nevi and plasma hormone levels in the plasma study in the Nurses' Health Study.

Number of Cutaneous Nevi	*N*	Estradiol (pmol/l)	Free Estradiol (pmol/l)	Testosterone (nmol/l)	Free Testosterone (nmol/l)	Dehydroepiandrosterone Sulfate (µmol/dl)	Sex Hormone–Binding Globulin (nmol/l)
None	380	28.60	0.40	0.75	0.007	1.81	64.45
1–2	169	30.25	0.44	0.75	0.008	1.93	57.53
3–5	49	33.08	0.48	0.81	0.008	1.92	66.19
6+	13	37.70	0.59	0.91	0.01	1.61	56.90
*p* for trend		0.02	0.001	0.06	0.001	0.65	0.50

Adjusted for fasting status (<8 h, ≥8 h), time of day (24-h clock: <8, 8–12, 13–24), age at blood draw (continuous), season of blood draw (May to October, other months), alcohol intake (continuous), smoking status (never, ever), and body mass index at blood draw (continuous).

We further conducted a case-control study among a subgroup of 362 breast cancer cases and 611 controls matched for plasma hormone levels to adjust for hormone levels in the association between nevi and breast cancer risk: the multivariable-adjusted odds ratio for every five nevi was attenuated from 1.25 (95% CI, 0.89–1.74) to 1.16 (95% CI, 0.83–1.64) after adjusting for plasma hormone levels. The hormones that we adjusted for were estradiol, testosterone, sex hormone–binding globulin, dehydroepiandrosterone sulfate, free estradiol, and free testosterone. These data suggest that the association between nevus count and breast cancer risk was at least partially mediated through hormone levels.

We further evaluated the association between the number of cutaneous nevi and breast cancer risk by restricting analysis to the subgroup of postmenopausal women without PMH use in our cohort, and detected an unchanged HR from that in the overall population (multivariable-adjusted HR, 1.08).

We also examined the interactions between the number of cutaneous nevi and other risk factors for breast cancer, and there was no significant interaction after adjusting for multiple comparisons (*p* for interaction = 0.38 for current body mass index, 0.39 for body mass index at age 18 y, 0.87 for height, 0.75 for physical activity, 0.01 for multivitamin use, 0.80 for smoking status, 0.97 for alcohol consumption, 0.21 for age at menarche, 0.79 for parity and age at first birth, 0.23 for history of benign breast disease, 0.85 for family history of breast cancer, 0.59 for menopausal status, and 0.38 for PMH use). Additionally, the stratification analysis by menopausal status showed significant associations between the number of cutaneous nevi and breast cancers for both pre- and postmenopausal women, with an HR of 1.20 (95% CI, 1.04–1.40) for premenopausal women and an HR of 1.07 (95% CI, 1.01–1.12) for postmenopausal women (*p* for interaction = 0.59; Table S2).

Previous studies have suggested a possible role of sun exposure/vitamin D on breast cancer prevention. Thus, we additionally adjusted for the UV flux, outdoor activity, hair color, and skin tanning ability in this study: the HR for breast cancer remained unchanged after the adjustment (multivariable-adjusted HR, 1.08, 95% CI, 1.03–1.13). No interaction was detected between these factors and nevus count after adjusting for multiple tests (*p* for interaction = 0.87, 0.89, 0.93, and 0.68 for UV flux at birth, age 15 y, age 30 y, and the current year, respectively; 0.62, 0.30, 0.07, and 0.04 for outdoor activity during high school and at ages 25–35, 36–59, and 60–65 y, respectively; 0.39 for hair color; and 0.39 for skin tanning ability).

## Discussion

In this large prospective cohort study, we identified a significant positive association between the number of cutaneous nevi and the incidence of invasive breast cancer. This association was independent of the previously known risk factors for breast cancer, and the risk increased with the number of cutaneous nevi in a dose–response relationship. We further found that postmenopausal women with more cutaneous nevi had higher levels of plasma total and free testosterone and estradiol, and that the number of cutaneous nevi was associated with increased risk of breast cancer only among ER-positive tumors, suggesting that a hormonal effect underlies this association.

Melanocytes have been postulated to exhibit some degree of sex hormone responsiveness [14]. Estrogen and androgen receptors have been found in melanocytes, and melanogenesis is responsive to these steroid hormones [15]. Taken together with the phenomenon that melanocytic nevi commonly darken and/or enlarge during pregnancy, our present finding that women with more cutaneous nevi had increased plasma levels of free androgen and free estrogen further supports the association between cutaneous nevi and sex hormones.

In addition, high levels of pre-diagnostic circulating estrogens and androgens are consistently associated with increased risk of breast cancer, especially among postmenopausal women [16]. Estrogens can bind to ERs and contribute to the growth of breast cancer, and androgens may act directly, promoting cellular growth and proliferation via binding to the androgen receptor [17], or indirectly, via their aromatization to estrogens, either peripherally or in breast tissue [18]. More recently, a nested case-control study of breast cancer within the NHS cohort found that higher plasma free testosterone and free estradiol levels were significantly associated with increased risk of breast cancer, and the stratification analysis by ER/PR status showed significant associations only among ER+ tumors [10]. Consistent with these findings, we found increased risk of breast cancer among women with more cutaneous nevi, and this association held only among ER+ tumors.

Our study has several strengths. First, this is a prospective cohort study with a large sample size and long follow-up, providing robust evidence of the association between the number of cutaneous nevi and breast cancer risk. Second, we updated assessments of previously identified risk factors for breast cancer periodically and thoroughly adjusted for these factors. Thus, we were better able to examine the association of breast cancer with the number of cutaneous nevi independent of previously known risk factors for breast cancer. Third, we identified the number of cutaneous nevi as a phenotypic marker for plasma sex hormone levels, which suggests a possible mechanism underlying the association of breast cancer with cutaneous nevi. In addition, a single plasma hormone measurement provides a reasonable measure of levels over multiple years and may predict breast cancer for up to 16–20 y [10]. PARs are often used to quantify the risk conferred by a modifiable risk factor, and while nevi are likely a marker rather than a risk factor, we calculated the PAR of nevi to compare them with breast cancer risk factors. We calculated a PAR of 2.0% for women with 1–5 nevi and 1.2% for women with six or more nevi. These are on the order of the PARs of modifiable risk factors for breast cancer reported in previous studies: 2.5%–5.6% for hormone use, 0%–6.1% for ≥2 alcoholic drinks daily, and 4.6%–11.0% for physical inactivity [19].

One limitation of this study is that the number of cutaneous nevi in our cohort was self-reported. However, the majority of studies on cutaneous nevi have shown a substantial agreement between nevus self-counts and dermatologist counts, as well as a high reproducibility [20]. In addition, we collected information on nevus count of nevi of ≥3 mm diameter size on the left arm to represent the whole body. Examining the upper limbs only was suggested previously to be a practical and suitable tool for predicting total nevus count [21]. The self-reported number of cutaneous nevi in our cohort predicted melanoma risk [22], and our genome-wide association study on self-reported number of cutaneous nevi in this cohort confirmed previously identified loci in nevogenesis [23]. Thus, although the number of cutaneous nevi was self-reported, these findings suggest it is a valid assessment.

In summary, we identified the number of cutaneous nevi as a novel phenotypic marker associated with breast cancer risk. Given that higher numbers of cutaneous nevi reflect higher levels of plasma free testosterone and free estradiol, the number of cutaneous nevi may be a surrogate for sex hormone exposure. Our study was conducted in white individuals, so further studies are needed to confirm our findings in other populations. In addition, because our study was observational, these results should be interpreted cautiously and are insufficient to alter current clinical recommendations. Nevertheless, our data provide epidemiological evidence on the possible link between cutaneous nevi and breast cancer risk and support a need for continued investigation of this relationship.

## Supporting Information

Checklist S1 **STROBE checklist.**
(PDF)

Table S1 **Baseline characteristics of women with missing and available information on the self-reported number of cutaneous nevi.**
(DOC)

Table S2 **The number of cutaneous nevi and breast cancer risk by menopausal status at diagnosis.**
(DOC)

## Abbreviations

CI: confidence interval
ER: estrogen receptor
HR: hazard ratio
NHS: Nurses' Health Study
PAR: population attributable risk
PMH: postmenopausal hormone
PR: progesterone receptor
