# Correction: Association between Cutaneous Nevi and Breast Cancer in the Nurses' Health Study: A Prospective Cohort Study

**DOI:** 10.1371/journal.pmed.1001797

**Published:** 2015-03-03

**Authors:** The PLOS Medicine Staff

There is a missing affiliation for the sixth author. Jiali Han should also be affiliated with #9: Department of Epidemiology, Tianjin Medical University Cancer Institute and Hospital, Tianjin, China.
