# Supplementary material for: Association between Cutaneous Nevi and Breast Cancer in the Nurses' Health Study: A Prospective Cohort Study
Source: PLoS Med. 2014 Jun 10;11(6):e1001659. doi: 10.1371/journal.pmed.1001659 (PMC4051600; doi:10.1371/journal.pmed.1001659)
Supplement: Table S1 — Baseline characteristics of women with missing and available information on the self-reported number of cutaneous nevi. (DOC) [file pmed.1001659.s002.doc]

Table S1. Baseline characteristics of women with missing and non-missing information on the self-reported number of cutaneous nevi.

| Characteristics | Information on the number of cutaneous nevi | |
| --- | --- | --- |
|  | missing (n=18,020) | Non-missing (n=74,523) |
| Body mass index, kg/m^2^ (SD) | 25.1(4.7) | 25.3(4.8) |
| Body mass index at age 18, kg/m^2^ (SD) | 21.3(3.0) | 21.4(3.0) |
| Height at 1976, m (SD) | 64.3(3.6) | 64.5(3.1) |
| Physical activity, met-h/week (SD) | 13.6(22.9) | 14.1(20.7) |
| Multi-vitamin use, % | 41.7 | 43.1 |
| Current smoker, % | 26.9 | 21.6 |
| Alcohol consumption, gm/day (SD) | 6.2(10.5) | 6.2(10.7) |
| Age at menarche, ≤12, % | 48.4 | 49.2 |
| Nulliparous, % | 6.0 | 6.7 |
| Postmenopausal women, % | 56.9 | 60.2 |
| Duration of menopause among postmenopausal women, years (SD) | 11.6(6.5) | 10.2(6.4) |
| Current hormone use among postmenopausal women, % | 25.6 | 27.2 |
| Age at first birth, years (SD) | 28.8(16.3) | 29.3(17.2) |
| History of benign breast disease , % | 12.8 | 15.6 |
| Family history of breast cancer, % | 8.6 | 10.5 |
| ER/PR status |  |  |
| ER+/PR+, % | 70.9 | 69.3 |
| ER+/PR-, % | 13.3 | 13.9 |
| ER-/PR+, % | 2.4 | 2.2 |
| ER-/PR-, % | 13.3 | 14.6 |

^1^ Based on information collected in the 1986 questionnaire unless specified.
