# Supplementary material for: Association between Cutaneous Nevi and Breast Cancer in the Nurses' Health Study: A Prospective Cohort Study
Source: PLoS Med. 2014 Jun 10;11(6):e1001659. doi: 10.1371/journal.pmed.1001659 (PMC4051600; doi:10.1371/journal.pmed.1001659)
Supplement: Table S2 — The number of cutaneous nevi and breast cancer risk by menopausal status at diagnosis. (DOC) [file pmed.1001659.s003.doc]

Table S2. The number of cutaneous nevi and breast cancer risk by menopausal status at diagnosis.

| Nevus counts | Postmenopausal | |  | Premenopausal | |
| --- | --- | --- | --- | --- | --- |
|  | Cases | Multivariable-adjusted HR (95% CI)^1^ |  | Cases | Multivariable-adjusted HR (95% CI)^1^ |
| None | 3,052 | Ref |  | 252 | Ref |
| 1-5 | 1,609 | 1.04 (0.98, 1.10) |  | 134 | 0.95 (0.77, 1.18) |
| 6-14 | 200 | 1.12 (0.97, 1.29) |  | 25 | 1.39 (0.91, 2.13) |
| 15+ | 53 | 1.32 (1.01,1.74) |  | 8 | 2.24 (1.08, 4.64) |
|  |  |  |  |  |  |
| HR for per 5 nevi | 4,914 | 1.07 (1.01, 1.12) |  | 419 | 1.20 (1.04, 1.40) |
| P for trend |  | 0.01 |  |  | 0.02 |
| *P* for interaction = 0.59 | | | | | |

^1^ Adjusted for age, age at menarche, parity and age at first birth , body mass index, body mass index at age 18, height, physical activities, multivitamin use, family history of breast cancer in a first-degree relative, cigarette smoking, alcohol consumption, self-report of benign breast disease, as well as duration of menopause and hormone use among postmenopausal women.
